# *Escherichia coli* Strains with Virulent Factors Typical for Uropathogens were Isolated from Sinuses from Patients with Chronic Rhinosinusitis—Case Report

**DOI:** 10.3390/pathogens9050318

**Published:** 2020-04-25

**Authors:** Beata Krawczyk, Michał Michalik, Magdalena Fordon, Magdalena Wysocka, Alfred Samet, Bogdan Nowicki

**Affiliations:** 1 Department of Molecular Biotechnology and Microbiology, Faculty of Chemistry, Gdańsk University of Technology, 80-233 Gdańsk, Poland; Magdalena.fordon@pg.edu.pl (M.F.); magwojta@student.pg.edu.pl (M.W.); 2 Medical Center MML, 00-112 Warsaw, Poland; michalim@mml.com.pl (M.M.); dr.alfredsamet@gmail.com (A.S.); 3 Nowicki Institute for Women’s Health Research (Now I for HeR), 114 Governors Way, Brentwood, TN 37027, USA; bnowicki1949@gmail.com

**Keywords:** biopsy, *E.coli*, virulence factors, UPEC, chronic rhinosinusitis

## Abstract

*Escherichia coli* were isolated from three patients with chronic rhinosinusitis (CRS) by intraoperative sinus tissue biopsy. Taking into account the unusual replicative niche and previous treatment failures, it was decided to focus on the virulence and drug resistance of these bacteria. The strains turned out to be multi-sensitive, but the rich virulence factors profile of bacteria typical for phylogenetic group B2 deserved attention. Tests were carried out for the presence of 32 genes using the PCR method. Particularly noteworthy are the toxins Cnf-1, HlyA, Usp—an extensive iron uptake system (enterobactin, salmochelin, yersiniabactin and outer membrane hemin receptor ChuA)—SPATE autotransporters such as *vat* and *pic,* Ag43 autoaggregative protein—important for biofilm formation—and TosA/B which enhance the fitness of *E.coli*. All these virulence factors are identified predominantly in UPEC strains and provide a fitness advantage during colonization of the sinuses. Patients with CRS should be asked for past or present UTI. The specific virulence factors of *E. coli* that facilitate the colonization of the GI tract and urinary tract may also favor the colonization of a new ecological niche (sinuses) as a result of microbial imbalance or dysbiosis.

## 1. Introduction

Chronic rhinosinusitis (CRS) is one of the most common chronic diseases in the European population according to published studies (GA2LEN CRS study) and ranges from 6.9% to 27.1%. There are known patient-associated risk factors, e.g., atopy, asthma, nasal polyposis, tissue remodeling, mucociliary clearance (MCC) disturbances, and deficiencies in the host immune response, while the nature of the interaction of microorganisms with the host remains largely unknown. It is unclear whether bacteria contribute to the development of infection, initiate an inflammatory response due to the production of superantigens, or whether facilitated sinus colonization is the result of changes in the sinus mucosa. The sino-nasal cavity has a resident flora that is responsible for the right environment for the respiratory tract. In case of sinusitis, conditions promote the growth of bacteria (temperature, humidity). To date, attention has been focused on traditional pathogenic microorganisms, e.g., *Streptococcus pneumoniae, Haemophilus influenzae, Moraxella catarrhalis, Stenotrophomonas maltophilia* and *Enterobacter* species. These bacteria also occur in healthy people in smaller amounts, but when the bacteria exceed 1000 Colony Forming Units (CFU) per milliliter of mucus, they are regarded as a pathogenic [1]. Due to long-term therapy with antibiotics, keystone species of microbiota are eliminated from natural environment. Imbalance or dysbiosis in CRS may result in a decrease in microbial diversity and microbiome disorder. Pathogenic bacteria can lead to a serious imbalance in the local microbiome and disrupt its function as an immunomodulator [2,3]. *Escherichia coli* has been documented as an associate etiological factor of chronic sinusitis [4,5] but the nature of the virulence of *E. coli* in chronic sinusitis remains largely unknown. Gene variability and modification of virulence gene expression affecting the process of bacterial adhesion to host cells. *E. coli* is characterized by high plasticity of the genome, easily acquires various genes encoding virulence factors to facilitate its colonization, invasion or overcoming the host’s immune barrier [6]. In these case reports, we analyzed CRS in association with bacterial risk factors.

## 2. Results

### 2.1. Patient’s Medical History 

#### 2.1.1. Case 1 Presentation

We present the case of a 41-year-old man with CRS (sinus pain, burning sensation and blocked nose, mucosal abnormalities, and nasal congestion). The patient has undergone surgery of the nasal septum and functional endoscopic sinus surgery. In the postoperative period, the patient had a dry discharge in the sinuses. After few weeks, the patient again felt a runny nose, as well as an unpleasant nose odor. Laryngological studies have confirmed the occurrence of changes in sinuses. Due to a lack of improvement, the patient qualified for another sinus surgery. Half a year later, a functional endoscopic sinus surgery was performed with the opening of tubular cells, frontal sinuses. Histopathological examination confirmed the presence of polypoid mucosal epithelial cells without squamous metaplasia, mononuclear infiltrates and eosinophilic granulocytes (eosinophils accounted for 30% of inflammatory mass). Microbiological studies of biopsy indicated polymicrobial infection including *E.coli*.

#### 2.1.2. Case 2 Presentation

A patient aged 44 years reported to the Medical Center because of nasal obstruction and snoring. The patient suspects sleep apnea. In otolaryngologic examination, the obstruction of the nasal septum and the lower nasal concha hypertrophy were confirmed. The patient also had a flabby, drooping and overly overgrown soft palate. Computed tomography confirmed the occurrence of inflammatory lesions in the maxillary and ethmoidal sinuses. Functional endoscopic nasal and nasal sinuses surgery, nasal septum correction, and correction of soft palate by coblation method were performed. Intraoperatively, hypertrophic edema of the sinus mucosa with mucous secretions was observed. Histopathological examination confirmed that the material was part of the sinus mucosa coated with cylindrical epithelium. Inflammatory infiltrates found in the stroma contained up to 30% of plasma cells and eosinophilic granulocytes. Bacteriological findings confirmed the presence of *E. coli* and *E. faecium*.

#### 2.1.3. Case 3 Presentation 

An 18-year-old man with a long-standing nasal obstruction complained of nasal congestion and bleeding. In otolaryngologic examination, the obstruction of the nasal septum and the lower nasal concha hypertrophy were observed. The otoscopic examination showed no changes in the ears. Computed tomography revealed polypoid lesions in the maxillary, ethmoid and frontal sinuses. Functional endoscopic sinus surgery as well as correction of nasal septum by septoplasty and correction of nasal conchae by coblation method were performed. Coblation (short for controlled ablation) is a modern surgical technique that uses the energy of electromagnetic waves to generate low-temperature plasma in a saline environment. Coblation allows for removal of tissue while preventing excessive bleeding at the tissue removal site. Intraoperatively a large amount of mucus was observed in the sinuses. Histopathological examination of the material revealed the presence of mucosal fragments of sinuses covered with cylindrical epithelium. Infiltrates of plasma cells, neutrophilic and eosinophilic granulocytes were present in the stroma. Eosinophil content on both sides reached 50% of inflammatory cells. Bacteriological studies confirmed the presence of *E.coli.*

Details related to the clinical and microbiological characteristics of the patient and bacteria are given in Table 1. 

### 2.2. Genetic Characterization of Escherichia coli 

The *E. coli* strains were subjects for genetic characterization (Table 2.). The B2 phylogenetic lineage of *E. coli* belong to highly pathogenic ExPEC. The presence of genes coding fimbrial and nonfimbrial adhesins, genes coding of toxins, genes coding of iron accuisition system, K capsule, invasion IbeA, autotransporters SPATE group and Ag43a (*flu*)—autotransporter and adhesin with function of biofilm formation—were detected. We also found *tos*A/B genes that enhance the fitness of *E.coli*.

## 3. Discussion

Antibiotic therapy targets usually classic pathogens associated with sinusitis. In the presence of Gram-negative pathogens, including *E. coli*, such therapy may result in therapeutic failure and/or a chronic infection. This study presents three clinical cases of patients with CRS not responsive to therapy. An intraoperative sinus tissue biopsy of these three patients provided us valuable information that sinuses microbiota of each patient contained *E. coli* (Table 1). The treatment accommodating *E. coli*, which is not typically detected in CRS, resulted in the resolution of CRS symptoms [4,5,7,8].

The results of genetic characterization of the virulence profile of *E. coli* strains isolated from the sinuses was consistent with the genetic profile of highly virulent pathogens in *E. coli* isolated from patients with CRS contained toxins typical for uropathogenic strains (UPEC): cytotoxic necrotizing factor 1, uropathogenic specific protein (Usp), and α-hemolysin. *E. coli* toxins are dangerous, enabling the release of nutrients from host cells, generating a fitness in an ecological niche and enabling bacteria to spread into the infected tissue [9]. 

We also detected several adherence profiles (1,3, S, P, F1C fimbriae) that are typical for highly pathogenic UPEC strains. These adhesins facilitate tissue colonization due to binding capacity to specific cell receptors expressed on epithelial cells [9]. In addition, detection of the *tos*A and *to*sB genes associated with the island of pathogenicity (PAI-aspV) suggest an enhanced fitness of UPEC [10,11].

There is not much known about receptor similarity between sinus epithelium and the epithelium in the urinary tract. However, detection of type 1, S and P fimbriae common in the *E. coli* group responsible for the UTI, meningitis, or the risk of sepsis may suggest such potential [6,12,13]. All three *E. coli* strains had the *ag*43a gene encoding of protein that is frequently involved in biofilm formation and it is an additional bacterial-associate risk factor [9]. Biofilm is responsible for maintaining the inflammation of sinuses and blocks the penetration of the antibiotic. Therefore, systemic antibiotics often do not reach the sinus area and sustain chronic infection [14]. 

**Figure 1:**
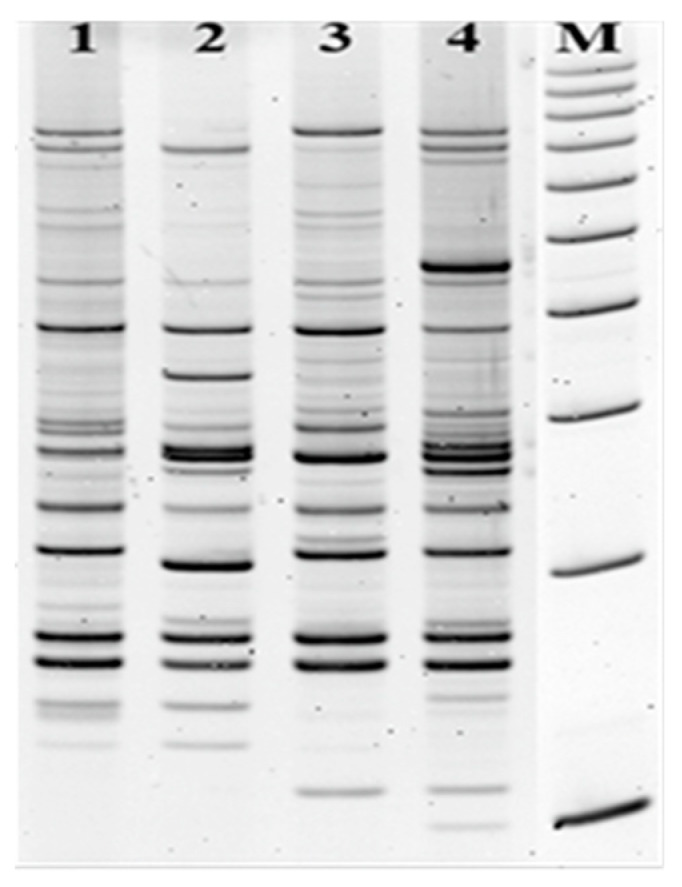
Analysis of genomic DNA of *E. coli* by LM-PCR/Shifter method. The genotypes indicate different fingerprint patterns for *E. coli* strains. 1. Pattern of UPEC clinical strain unrelated with patients; 2. pattern for *E. coli* strain isolated from Patient I; 3. pattern for *E. coli* strain isolated from Patient II; 4. pattern of *E. coli* strain from Patient III. M- the molecular DNA size marker (100–1000 bp).

Serine protease autotransporters (SPATEs) have proteolytic activity to erythroid spectrin, mucin, pepsin, and human coagulation factor V. There is an interesting occurrence of *vat* and *pic* genes for all isolates. Vat is the vacuolating autotransporter toxin recently described for avian pathogenic *E. coli* (APEC) and for UPEC strains [15,16]. Another gene, *ibe*A, was detected for *E. coli* isolated from patient I. IbeA invasion mediates interaction with intestinal epithelia and macrophages but also is responsible for recognition of receptors on the surface of brain endothelial cells and may lead to meningitis [9,17]. The proximity of the sinuses and the brain creates potential risk for the patient. Most importantly, several iron-acquisition systems were identified in tested strains. Bacteria have several mechanisms of acquiring the iron from the host and regulating during bacterial infection decrease of iron-binding proteins such as lactoferrin or transferrin. Lipokalin- 2 (LNC2) blocks an enterobactin (Ent) in acquiring iron from host cells (sequestration) but iron uptake by other siderophores can provide a competitive advantage and allows the colonization of an unusual bacterial niche. Siderophores modulate the microflora composition and regulate the growth of individual bacteria. They also may induce inflammation and may affect the spread of bacteria during sinusitis. All three *E.coli* isolates carried enterobactin (*entB, fepA*), yersiniabactin (*irp*2, *fyu*A) and salmochelin (*iro*N) genes; only aerobactin (*iuc*A,*iut*A) was absent. The salmochelin and yersiniabactin are not recognized by Lcn2; hence, they are important to bacterial growth and correlate with enhanced virulence. ChuA as an outer membrane hemin receptor is decisive for belonging to the high pathogenic B2 phylogenetic group of *E. coli* and was common for all isolates. These genes shall be treated as the markers of virulence [18]. All three *E. coli* strains were susceptible to antibiotics, indicating that the strains were not hospital acquisitions. The comparative analysis of electrophoretic patterns indicates that strains isolated from three patients are not closely related consistent with non-hospital origin (Figure 1).

Treatment for *S. aureus* (for case 1 and 3) and *E. faecalis* (for case 2) did not provide complete relief from the symptoms of CRS until to introduction the direct antibiotic therapy focused on *E. coli*. Controlled otolaryngologic examination confirmed that nasal patency is very good, without signs of bleeding or infection in the upper respiratory tract. It suggests that detection of *E. coli* as a result of microbiological tests should not be underestimated.

We consider that the appearance of UPEC strains in the sinuses may suggest their origin from the patient’s digestive or urinary tract and their transmission to a new ecological niche. This could be a result of long-term antibiotic therapy, which leads to dysbiosis in the patient’s body. Understanding the fitness strategies employed by *E. coli* during colonization/infection of a rhinosinusitis could be useful for novel developing intervention strategies.

## 4. Materials and Methods 

### 4.1. Patients and Samples

In this study of three patients with chronic sinusitis, the histopathology of the biopsied sinus mucosal lining, endoscopy and computed tomography were performed at the Medical Center MML in Warsaw, Poland. All subjects gave their informed consent for inclusion before they participated in the study. The study was conducted in accordance with the Declaration of Helsinki, and the protocol was approved by the Ethics Committee of at the Medical University of Lodz RNN/ 128/17/ EC dated 11.04.2017. 

Specimens were collected by doctors during the FESS procedure for transport media and delivered to the microbiology laboratory within three hours. After cultivation, bacteria were kept in -80°C at a cryobank. The collection procedures included minimizing the risk of contamination. The semiautomatic bioMerieux Vitek2 analyzer was used for bacterial identification and antibiotic susceptibility testing. In addition, mycological cultures for yeast-like fungi and for anaerobic bacteria were negative in all three cases. Patients were subjected to an additional interview to determine any previous urinary tract infections (Table 1). 

### 4.2. Genetic Characterization of E. coli Strains 

The extraction of total DNA from bacterial liquid cultures with *Extractme DNA Bacteria Kit* (BLIRT S.A. Gdansk, Poland) according to the manufacturer’s procedure. Genomic fingerprints by LM PCR/Shifter was used to study relationships between isolates to reject contamination or nosocomial infection. The details of the method have been described by Krawczyk et al. [19,20]. This method is based on the digestion of total bacterial DNA with one restriction enzyme, FokI (Class IIS), and ligation with oligonucleotide adapters (with NGCN 5’ end sequence), and PCR with NGCN 3’ end sequence of primer. The method does not require prior knowledge of the sequence of the analyzed DNA and generates a limited number of DNA fragments, whose band pattern on the gel differs between strains of a bacterial species. DNA fragments were easily analyzed on 6% polyacrylamide gels stained with ethidium bromide (Figure 1). The patterns that were differ by greater than three bands were regarded as unrelated. 

The assignment of *E. coli* isolates to the phylogenetic group B2 was done based on quadruplex phylo-typing PCR assay for five DNA markers (*chu*A, *yja*A, the DNA fragment TSPE4.C2, *arp*A and *trp*A) as described in Clermont et al. [18]. 

The virulence factors such as type 1 fimbriae (*fim*G/*fim*H), S fimbriae (*sfa*D/*sfa*E), P fimbriae (*pap*C), F1C fimbriae (*foc*G), type 3 fimbriae (*mrk*D), family fimbrial and afimbrial adhesins Afa/Dr (*afa*/*dr*), α-hemolysin (*hly*A), cytotoxic necrotizing factor 1 (*cnf*1), uropathogenic specific protein (*usp*), synthesis capsule (*ksp*MTII), enterobactin Iha-iron-regulated gene homologue adhesion (*iha*), aerobactin receptor (*iut*A), yersiniabactin receptor (*fyu*A), invasion of brain endothelium A (*ibe*A) and the *ag*43 gene responsible for the biofilm formation with autotransporter protein function simultaneous, were detected by multiplex or simplex PCR as described previously [6,13,21,22]. For other genes, we used the same profile of PCR but a different annealing temperature as follows: 95 ℃ for 120 s in one cycle, for 30 cycles: 95 ℃ 30 s, 64 ℃ for *tos*B/*tos*A (ABC-transporter protein of the HlyB family/RTX family member nonfimbrial adhesins and toxin TosA); 55.5 ℃ for *ent*B (enterobactin synthesis gene) and for *iro*N (salmochelin receptor); 60 ℃ for *fep*A (enterobactim receptor) and for *fec*A (ferric citrate outher membranę transporter); 49.5 ℃ for *irp*2 (yersiniabactin gene synthesis); 50.5 ℃ for *iuc*A (aerobactin synthesis gene); 65.5 ℃ for *aida* (autotransporter), for 30 s and 72 ℃ for 45 s. Serine protease autotransporters (SPATE)—*vat, pic, sat, pic*-like (U), *pssA, boa, hbp* were detected by PCR/RFLP (HaeIII) as previously described [23] with some modification (T*a* in PCR was 60 ℃). Amplified products were run on 1.2%–1.5% agarose gels and visualized with ethidium bromide. Only PCR products after HaeIII restriction digestion (PCR-RFLP method) products were detected on 12% polyacrylamide gel and staining with ethidium bromide.

## 5. Conclusions

Establishing the *E. coli* phylogenetic group (commensal/pathogenic strains) and their virulence factors profile may facilitate a better understanding of the various pathophysiological aspects related to sinus colonization by *E. coli* and the development of sinusitis; therefore, their origin could be either in the urinary or digestive tract. In conclusion, the diagnosis and treatment of CRS should include interdisciplinary approach, intraoperative biopsy and the microbiological consultations. Extensive strain characterization with in-depth patient diagnostics may allow selection of more effective therapy. 

## Figures and Tables

**Table 1:** Clinical and microbiological characteristics of patients.

Clinical Characteristic of Patients	Case 1	Case 2	Case 3
**Basic demographics**			
Age	41	44	18
Gender	M	M	M
Underlying diseases	chronic sinusitis	chronic sinusitis	chronic sinusitis
Duration of illness	7 years	3 years	5 years
Duration of symptoms	>12 weeks	>12 weeks	>12 weeks
Episodes within a one-year period	>4	>4	>4
**Symptoms**			
Purulent drainage	+	+	+
Facial/dental pain	+	+	+
Nasal obstruction	+	+	+
Hyposmia	+	+	+
Nasal congestion	+	−	+
Halitosis	+	−	-
Cough, sore throat	+	+	+
Ear pain	−	−	+
**Patient-associated risk factors**			
Nasal polyps	+	−	+
Lower nasal concha hypertrophy	−	+	+
Cigarette smoking: Currently (C)/in the past (P)	+ (P)	+ (P)	+ (P)
Sleep apnea	-	+	-
Allergy	+	-	-
Snoring	−	−	−
GERD	−	−	−
^*^ Outpatient procedures within 6 months in violation of tissue integrity	dental treatment	-	-
Surgery	functional endoscopic sinus surgery	functional endoscopic nasal and nasal sinuses surgery, nasal segmental correction, correction of soft palate by coblation method	functional endoscopic sinus surgery, correction of nasal septum and conchae by coblation method
	CBCT 3D	CBCT 3D	CBCT 3D
Radiodiagnostics			

**Clinical outcome**			
Relapse	+	−	−
Duration of clinical remission	6 months	−	−
**Postoperative period**	functional endoscopic sinus surgery with the opening of ethmoid cells, frontal sinuses	−	−
**Histopathological data**			
Polypoid mucosal	+	−	+
Cylindrical epithelium	−	+	+
Infiltration with monocytes	−	+	+
Metaplasia	−	−	−
Blood count/NEU% [range 45–70]	64	51.7	51.5
Blood count/EOS% [range 1–5]	4.9	2.7	6.3
Blood count/MONO% [range 3–8]	9.4	8.5	5.4
Clinical sample	specimen/swab	specimen/swab	specimen/swab
**Microbiological cultures - characteristic**			
Polymicrobial infections	*E. coli; S. aureus*	*E. coli; E. faecium*	*E. coli; S. aureus*
Empiric Therapy	AMC	AMC	AMC
Inflammation of the urinary system	+	+	+
Episodes/ recurrence of infection	>10/+	2/−	1/−

Legend: AMC, amoxycillin/clavulanic acid; EOS%, percentage of eosinophils; NEU%, percentage of neutrophils; MONO%, percentage of monocytes; GERD, Gastroesophageal reflux disease; M, male; CBCT 3D - Cone beam computed tomography. * This item presents all outpatient procedures within 6 months related to violation of tissue continuity, minor surgical, dental, aesthetic medicine procedures, implants, ear piercing, etc.

**Table 2:** Genetic characteristic of *E. coli* isolates.

Genetic Characteristic of Isolates	Isolate 1	Isolate 2	Isolate 3
*Escherichia coli*	from left maxillary sinus	from right maxillary sinus	from left maxillary sinus
Antibiotic resistance profile of *E. coli*	-	AM, AMC, P, AMX, TRC, TR	-
Virulence -associated genes of *E. coli*			
Adhesins (fimbrial and nonfimbrial adhesin)	*fim*G/H, *sfa*, *pap*C, *tos*A	*fim*G/H, *sfa*, *foc*G	*fim*G/H, *sfa*, *foc*G
Toxins	*hly*A, *usp*, *cnf*1, *tos*B	*hly*A, *us*p, *cnf*1, *tos*B	*hly*A, *us*p, *cnf*1, *tos*B
Iron acquisition system	*fyu*A, *ir*p2, *en*tB, *fep*A, *iro*N, *chu*A	*fyu*A*, irp*2, *ent*B, *fep*A, *iro*N, *ih*a, *chu*A, *fec*A	fyuA, irp2, *ent*B, fepA, *iro*N, *iha*, *chu*A, *fec*A
K capsule	*ksp*MTII	*ksp*MTII	*ksp*MTII
Autotransporter and biofilm	*ag*43a (*flu*)	*ag*43a (*flu*)	*ag*43a (*flu*)
Serine protease autotransporters	*vat*, *pic*	*va*t, *pic*	*vat*, *pic*
Invasin	*ibe*A	-	-
*E. coli* phylogenetic group	B2	B2	B2

Legend: AM: ampicillin; AMC: amoxycillin/clavulanic acid; P: piperacillin; AMX: amoxycillin; TR: ticarcillin; TRC: ticarcillin/clavulanic acid; *fim*G/*fim*H, type 1 fimbriae; *sfa*D/*sfa*E, S fimbriae; *pap*C/G, P fimbriae; *foc*G, F1C fimbriae; *hly*A, α-hemolysin; *cnf*1, cytotoxic necrotizing factor 1; *usp*, uropathogenic specific protein; *ksp*MTII, synthesis capsule; *iha*, enterobactin *iha*-iron-regulated gene homologue adhesion; *fyu*A, yersiniabactin receptor; *irp2*, yersiniabactin gene synthesis; *fep*A, enterobactin receptor; *ent*B, enterobactin gene synthesis; *iro*N, salmochelin receptor; *chu*A, outer membrane hemin receptor; *fec*A, ferric citrate outher membranę transporter; *ibe*A, invasion of brain endothelium A; *ag*43a, biofilm formation with autotransporter protein function simultaneous; *vat*, vacuolating autotransporter toxin; *pic,* serine protease autotransporter; *tos*A, nonfimbrial adhesin; *tos*B, ABC-transporter protein of the HlyB family; *ibe*A, invasion, macrophage survival, inflammatory response.
